# Synkinesis assessment in facial palsy: validation of the Dutch Synkinesis Assessment Questionnaire

**DOI:** 10.1007/s13760-015-0528-7

**Published:** 2015-09-16

**Authors:** Ingrid J. Kleiss, Carien H. G. Beurskens, Peep F. M. Stalmeier, Koen J. A. O. Ingels, Henri A. M. Marres

**Affiliations:** Department of Otolaryngology and Head and Neck Surgery, Radboud University Medical Center, PO Box 9101, 6500 HB Nijmegen, The Netherlands; Department of Orthopedics, Section Physical Therapy, Radboud University Medical Center, Nijmegen, The Netherlands; Department for Health Evidence, Radboud University Medical Center, Nijmegen, The Netherlands

**Keywords:** Facial palsy, Synkinesis, Assessment, Synkinesis Assessment Questionnaire, Translation, Validation

## Abstract

The objective of this study is to validate an existing health-related quality of life questionnaire for patients with synkinesis in facial palsy for implementation in the Dutch language and culture. The Synkinesis Assessment Questionnaire was translated into the Dutch language using a forward–backward translation method. A pilot test with the translated questionnaire was performed in 10 patients with facial palsy and 10 normal subjects. Finally, cross-cultural adaption was accomplished at our outpatient clinic for facial palsy. Analyses for internal consistency, test–retest reliability, and construct validity were performed. Sixty-six patients completed the Dutch Synkinesis Assessment Questionnaire and the Dutch Facial Disability Index. Cronbach’s *α*, representing internal consistency, was 0.80. Test–retest reliability was 0.53 (Spearman’s correlation coefficient, *P* < 0.01). Correlations with the House-Brackmann score, Sunnybrook score, Facial Disability Index physical function, and social/well-being function were −0.29, 0.20, −0.29, and −0.32, respectively. Correlation with the Sunnybrook synkinesis subscore was 0.50 (Spearman’s correlation coefficient). The Dutch Synkinesis Assessment Questionnaire shows good psychometric values and can be implemented in the management of Dutch-speaking patients with facial palsy and synkinesis in the Netherlands. Translation of the instrument into other languages may lead to widespread use, making evaluation, and comparison possible among different providers.

## Introduction

Patients with facial palsy experience several problems; brow ptosis, incomplete eye closure (leading to exposure keratopathy), nasal valve collapse, oral incompetence, articulation difficulties, overall facial asymmetry, and psychosocial problems. Facial nerve injury and recovery is often accompanied with secondary effects; crocodile tears, eye dryness, taste disturbances, and synkinesis. Synkinesis is the phenomenon of involuntary movement in one (or more) area(s) of the ipsilateral face during voluntary movement in another area of the face. For example, eye closure during speaking or eating. Three possible mechanisms for the development of synkinesis are described. The first, and most widely accepted, proposed mechanism is that of aberrant regeneration. During regeneration axons might regrow in endoneural tubes other than their original ones, innervating different muscle groups. A second mechanism is the stimulation of neighbor axons due to loss of myelin. A last possibility is hyper excitability of the facial nucleus itself [1].

Patient-reported outcome measures and disease-specific quality of life have become more and more important, leading to the development of self-assessment questionnaires. Many grading scales for facial function have been developed, only a few including the assessment of synkinesis, for example the Sunnybrook facial grading system [2]. Assessment can be performed by the physician using clinician-based grading scales, but quantitative (sometimes automated) tools have been developed as well. The Synkinesis Assessment Questionnaire (SAQ) is a valid, reliable, and easily administered instrument for the self-assessment of synkinesis in patients with facial palsy [3]. This instrument was developed and validated in the Facial Nerve Center at the Massachusetts Eye and Ear Infirmary (Boston, USA) in 2007. The questionnaire consists of nine items. Total scores range from 0 (no synkinesis) to 100 (severe synkinesis, all the time). In our clinic, there was need for a synkinesis self-assessment tool and we wanted to be able to compare our results with other clinics.

The aim of this study was to create a Dutch version of the Synkinesis Assessment Questionnaire and to test its internal consistency, test–retest reliability, and construct validity for a valid use in the Dutch language and culture.

## Methods

### Translation

**Fig. 1:**
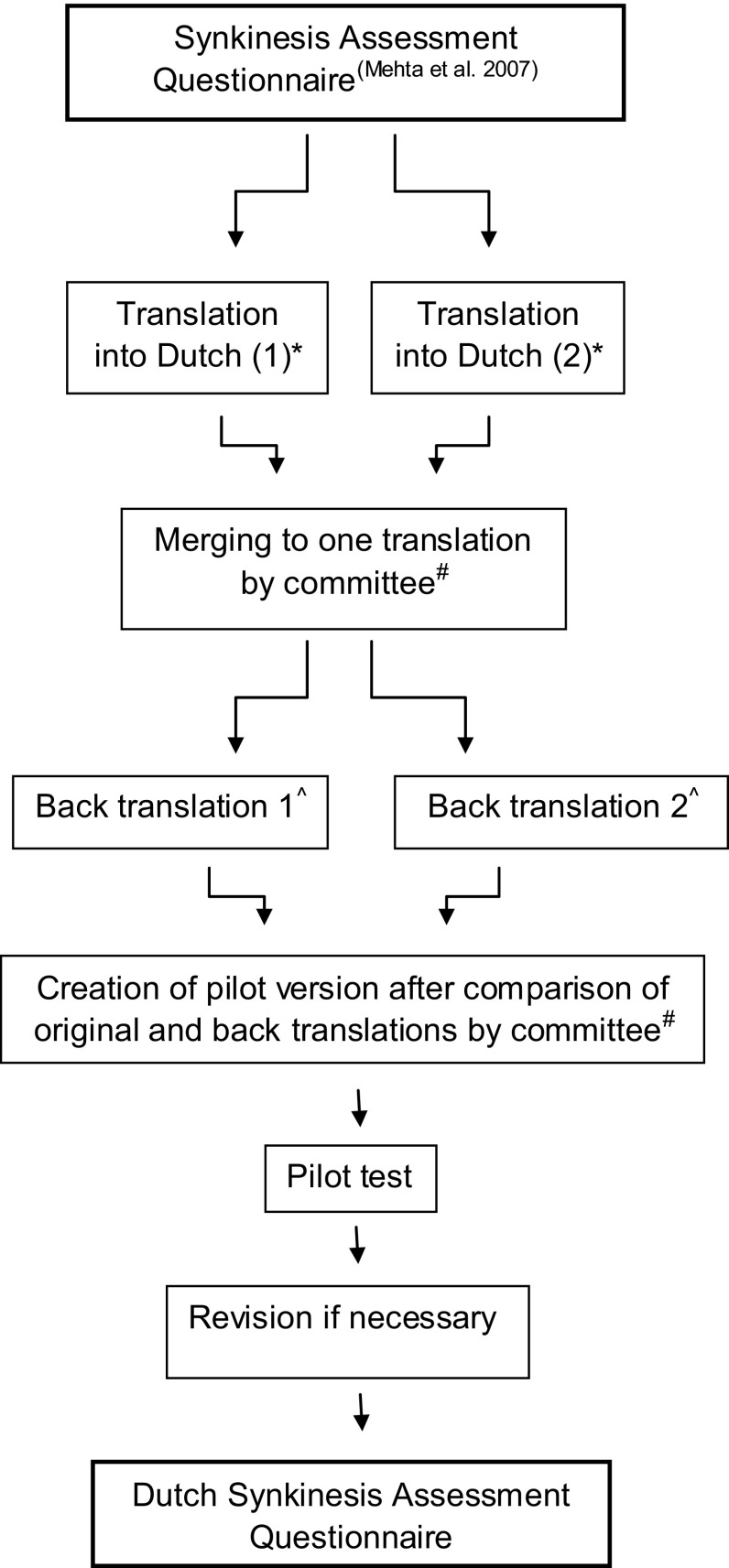
Method of forward–backward translation. *Asterisk* two independent translators; both native Dutch with American-English as their second, fluent, language; one of them was a medical doctor. *Hash* committee consisting of the authors of this manuscript. *Superscript* two independent translators; both American from origin with Dutch as a second language; one of them had a medical background

We approached the developers of the SAQ and obtained permission to use the instrument for translation and validation [3]. In the current literature, there is no consensus on ‘gold standard’ guidelines for translating quality of life questionnaires. Two methods are described; the forward–backward translation [4–6] and the dual-panel translation [7]. Dual-panel translation compromises the translation by a team of translators working together and assessment of the translation by a lay panel [8]. The forward–backward translation seems to be the most accepted method; although there is no evidence to support this view. Acquadro et al. performed a literature review in 2008; they did not find evidence in favor of one method, but strongly advised researchers to adopt a multistep approach [8]. A forward–backward translation approach was used in this study (Fig. 1). Considerations and difficulties of each step were documented. Choice of wording and phraseology had to be compatible with a reading level of age 14 [4]. The pilot test was performed in a group of ten patients with a facial palsy and ten persons without history of facial disease. Respondents completed the questionnaire and were asked about difficulties with answering and understanding the items. After this pilot test, final adjustments were made and documented.

### Validation

When using a questionnaire in another country and another language, translation of the items alone is not enough. The items must be adapted to the new culture to maintain the content validity of the instrument: cross-cultural adaption is required. [9, 10] Validation of the Dutch SAQ was performed at our university medical center between December 2012 and August 2014. The study protocol was assessed according to guidelines of the local committee on research involving human subjects; no formal ethical review was required. Dutch-speaking adult (18 years or older) patients with a facial palsy were included. Patients completed two different questionnaires: the Dutch SAQ and the Dutch Facial Disability Index (FDI). In addition, gender, age, etiology, side and duration of the palsy, House-Brackmann (HB) scores [11], and Sunnybrook (SB) scores [12] were collected in the database. This information was retrieved from the medical charts retrospectively.

To assess test–retest reliability, patients not receiving any form of treatment were sent the Dutch SAQ again after 2 weeks. At the end of the study, to increase the response rate for test–retest, patients were sent the SAQ (plus FDI) 2 weeks before visiting our clinic and the retest assessment was performed independently just before their visit while sitting in the waiting room. To test construct validity, the HB, SB, and FDI were used.

### Facial disability index

The FDI is a disease-specific quality of life questionnaire for patients with facial palsy, developed in at the Facial Nerve Center in Pittsburg around 1996 by VanSwearingen et al. [13]. The FDI has two domains; physical function and social/well-being function. The physical function scores range from −25 (worst) to 100 (best), and the social/well-being function scores range from 0 (worst) to 100 (best). The questionnaire does not have a synkinesis specific sub domain. This questionnaire has been translated into Dutch according to a forward–backward method previously (not published), but has not officially been validated for use in the Dutch culture.

### Statistical analysis

IBM SPSS Statistics 20 (IBM Corp. Armonk, NY) was used for data collection and statistical analysis. All questionnaire items were entered according to the principle of double data entry. First, descriptive analyses were performed to show patient characteristics. Cronbach’s *α* coefficient was calculated to test the internal consistency of the SAQ. Spearman’s correlation coefficient was calculated to analyze test–retest reliability. Correlations between the Dutch SAQ and the HB score, SB score, and FDI were calculated using Spearman’s correlation coefficient to show construct validity [14].

## Results

### Pilot test

Ten normal subjects, without history of facial disease, completed the pilot version of the Dutch SAQ, they all had a SAQ score of 0 (best score). Ten patients with peripheral facial palsy completed the pilot version of the translated questionnaire as well. Subjects did not document any difficulties in understanding or answering the items, one minor adjustment was made in the Dutch SAQ.

### Validation

**Fig. 2:**
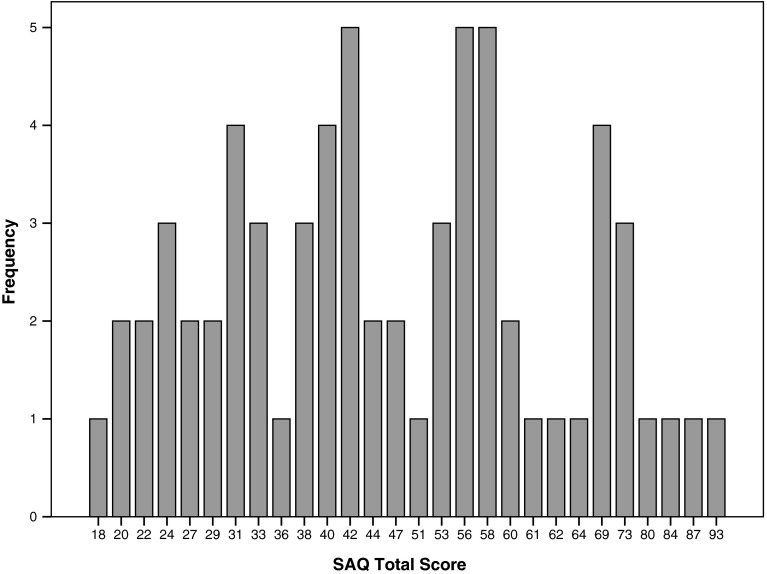
Skewness of SAQ scores

Between December 2012 and September 2014 66 patients completed the Dutch SAQ and FDI. Patient characteristics are shown in Table 1. Skewness of the SAQ scores is shown in Fig. 2, a small floor effect is seen.

**Table 1:** Patient characteristics

	*n*	%	Mean	SD	Median	Range
Gender						
Female	45	68				
Male	21	32				
Age (years)			55.8	13.4	57.5	25–89
Side						
Left	27	41				
Right	37	56				
Bilateral	2	3				
Time since onset (months)			40	50	26	5–300
Etiology						
Bell’s palsy	36	55				
Ramsay hunt	15	23				
Iatrogenic	3	5				
Traumatic	3	5				
Acoustic neuroma	3	5				
Other^#^	6	9				
House-Brackmann			3.0	0.9	3.0	1–5
Sunnybrook			47.9	17.7	49.5	11–83
FDI physical function			55.0	17.4	55.0	25–100
FDI social/well-being function			70.1	18.0	72.0	30–100
SAQ total score			47.6	18.0	44.4	18–93

^#^ Other etiologies comprised Lyme disease, cholesteatoma, multiple sclerosis, and benign facial nerve tumors

The internal consistency of the Dutch SAQ was assessed using Cronbach’s *α*, which showed a value of 0.80. A Cronbach’s *α* > 0.7 is generally considered acceptable, and *α* > 0.8 as good [14]. Test–retest reliability was moderate (0.53, Spearman’s correlation coefficient, *n* = 46, *P* < 0.01). Table 2 shows the individual item and total correlation coefficients.

**Table 2:** Individual item correlation coefficient (Spearman)

Item	Correlation	95 % CI
1	0.448	0.142–0.699
2	0.596	0.337–0.878
3	0.677	0.459–0.896
4	0.606	0.447–0.933
5	0.518	0.290–0.809
6	0.364*	0.127–0.696
7	0.447	0.214–0.813
8	0.522	0.265–0.794
9	0.396	0.073–0.667
Total	0.534	0.291–0.805

*CI* confidence interval

* Significant at the 0.05 level, all other items and total significant at the 0.01 level

Correlations between the SAQ score and the HB, SB, and FDI scores are shown in Table 3. Correlation with the HB score is negative because of the design of the HB (1 is no palsy, 6 is complete flaccid palsy).

**Table 3:** Correlation between SAQ and House-Brackmann, Sunnybrook scores, and Facial Disability Index (Spearman’s correlation coefficient, 95 % Confidence Interval)

	House-Brackmann	Sunnybrook	Sunnybrook synkinesis score	FDI physical function	FDI social/well-being function
SAQ Total score	−0.288	0.203	0.651**	−0.290*	−0.320*
	(−0.788 to −0.031)	(−0.110 to 0.560)	(0.199 to 0.834)	(−0.541 to −0.049)	(−0.547 to −0.039)

*SAQ* Synkinesis Assessment Questionnaire, *FDI* Facial Disability Index

* *P* < 0.05, ** *P* < 0.01

## Discussion

In this study, the Synkinesis Assessment Questionnaire has been translated and validated for use in the Netherlands. Cronbach’s *α*, representing internal consistency, was good (0.80). Test–retest reliability was moderate (0.53). Correlation with the Sunnybrook synkinesis sub score was moderate as well (0.50). Translation of the SAQ into the Dutch language and validation for use in the Dutch culture were performed according to the highest standards for translation of self-assessment questionnaires [5, 10, 15].

### Comparison with original questionnaire

Patient characteristics of this validation study are comparable with the original validation of the Synkinesis Assessment Questionnaire. Although, patients were on average about 10 years older. Bell’s palsy was the diagnosis in the majority of patients in both studies [3]. Internal consistency, reflected by Cronbach’s alpha coefficient, was 0.80 for the Dutch Synkinesis Assessment Questionnaire, in comparison to 0.86 for the original instrument. Test–retest reliability for the Dutch SAQ was moderate (0.53) in comparison to good (0.88) in the original validation study. Nevertheless, test–retest reliability was statistically significant for all items individually and for the total score in the current validation study (Table 2), overall with lower correlations than Mehta et al. [3].

### Comparison with other grading systems

The patient-reported SAQ scores showed low correlations with the clinician-based grading scales (HB and SB, −0.29 and 0.20, respectively). One reason for a low association with the HB is that the HB facial grading system does not take into account synkinesis. For instance, one patient in this study had a House-Brackmann score of 1, which represents normal facial function, while he did experience synkinesis. A higher correlation was demonstrated between the (clinician-based) Sunnybrook synkinesis sub score and the SAQ, *r* = 0.50. However, Mehta et al. found an even higher correlation between the SAQ and the Sunnybrook synkinesis sub scores (0.77). We sought for an explanation for this difference, but unsuccessfully. In any case, the SAQ is a patient-reported measure, while the HB and SB are physician-reported measures. Therefore a high correlation between these two types of measures is not expected. For this reason, both types of measures should be used in the assessment of synkinesis.

### Responsiveness

Future studies should demonstrate the responsiveness of the questionnaire. SAQ scores before and after treatment with botulinum toxin should be assessed, together with a clinician-based grading scale (Sunnybrook) and a quantitative, computerized tool. A study using all types of assessment will give us more insight in the responsiveness of the Dutch SAQ and the effectiveness of synkinesis treatment.

## Conclusion

The Synkinesis Assessment Questionnaire is a simple instrument for use in daily clinic. This subjective instrument should be combined with a quantitative instrument and clinician-based grading for a complete assessment of synkinesis. The use of the Dutch SAQ can now be implemented in the management of patients with facial palsy in the Netherlands. Implementation worldwide would facilitate comparison between clinics.

## Figures and Tables

**Fig. 3:**
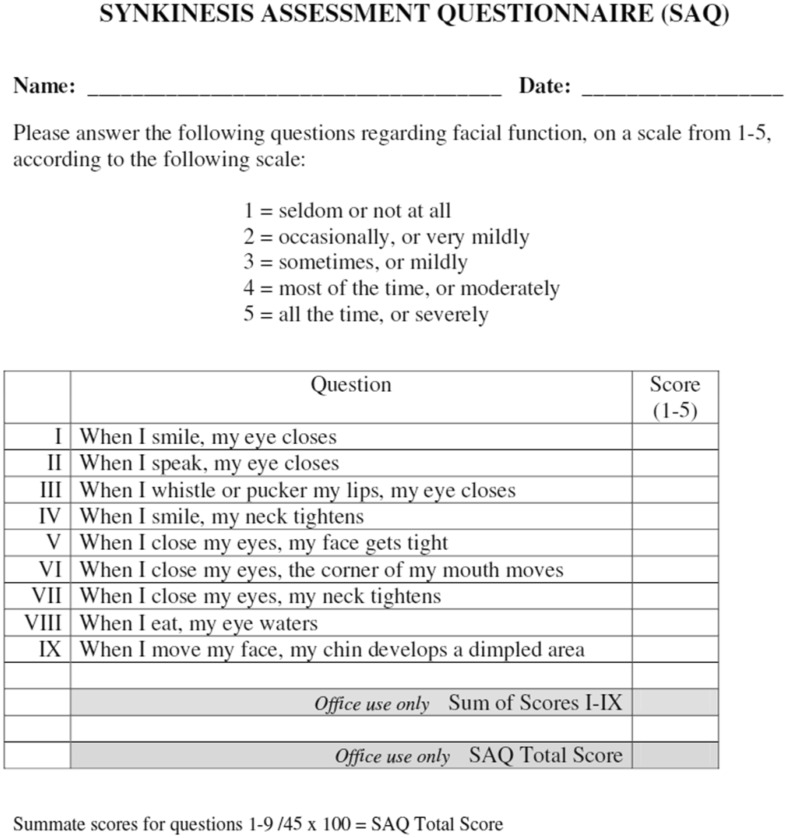
Original Synkinesis Assessment Questionnaire [3]

**Fig. 4:**
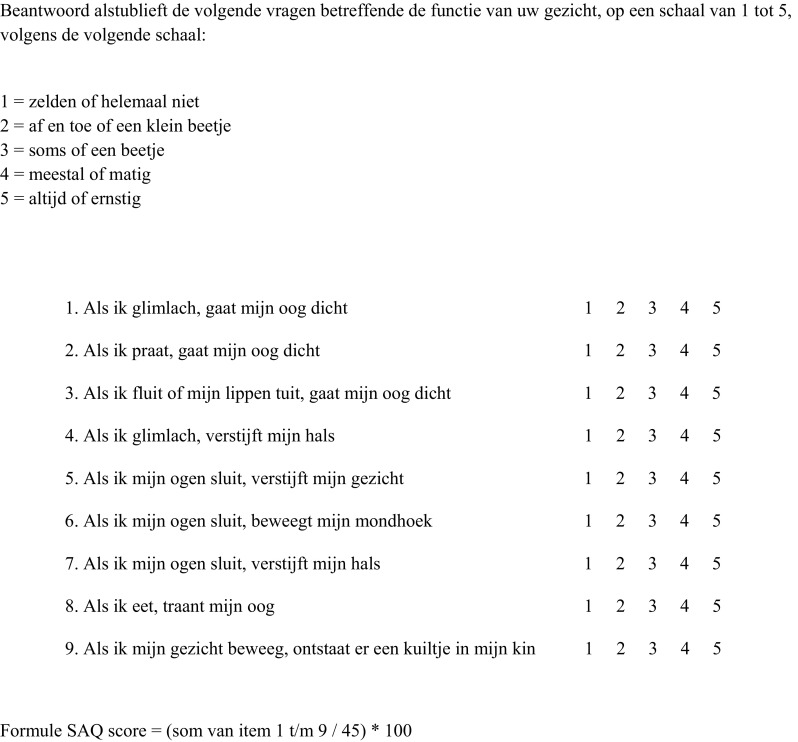
Dutch Synkinesis Assessment Questionnaire

## Appendix

See Figs. [3, 4].

## References

[1] Husseman J, Mehta RP (2008). Management of synkinesis. Facial Plast Surg.

[2] Ross BG, Fradet G, Nedzelski JM (1996). Development of a sensitive clinical facial grading system. Otolaryngol Head Neck Surg.

[3] Mehta RP, WernickRobinson M, Hadlock TA (2007). Validation of the Synkinesis Assessment Questionnaire. Laryngoscope.

[4] Bullinger M, Alonso J, Apolone G, Leplege A (1998). Translating health status questionnaires and evaluating their quality: the IQOLA Project approach. International Quality of Life Assessment. J Clin Epidemiol.

[5] Koller M, Aaronson NK, Blazeby J, Bottomley A (2007). Translation procedures for standardised quality of life questionnaires: the European Organisation for Research and Treatment of Cancer (EORTC) approach. Eur J Cancer.

[6] Wild D, Grove A, Martin M, Eremenco S (2005). Principles of Good Practice for the Translation and Cultural Adaptation Process for Patient-Reported Outcomes (PRO) Measures: report of the ISPOR Task Force for Translation and Cultural Adaptation. Value Health.

[7] Swaine-Verdier A, Doward LC, Hagell P, Thorsen H (2004). Adapting quality of life instruments. Value Health.

[8] Acquadro C, Conway K, Hareendran A, Aaronson N (2008). Literature review of methods to translate health-related quality of life questionnaires for use in multinational clinical trials. Value Health.

[9] Guillemin F, Bombardier C, Beaton D (1993). Cross-cultural adaptation of health-related quality of life measures: literature review and proposed guidelines. J Clin Epidemiol.

[10] Beaton DE, Bombardier C, Guillemin F, Ferraz MB (2000). Guidelines for the process of cross-cultural adaptation of self-report measures. Spine.

[11] House JW, Brackmann DE (1985). Facial nerve grading system. Otolaryngol Head Neck Surg Off J Am Acad Otolaryngol Head Neck Surg.

[12] Ross BG, Fradet G, Nedzelski JM (1996). Development of a sensitive clinical facial grading system. Otolaryngol Head Neck Surg Off J Am Acad Otolaryngol Head Neck Surg.

[13] VanSwearingen JM, Brach JS (1996). The Facial Disability Index: reliability and validity of a disability assessment instrument for disorders of the facial neuromuscular system. Phys Ther.

[14] Field A (2005). Discovering statistics using SPSS.

[15] Guillemin F, Bombardier C, Beaton D (1993). Cross-cultural adaptation of health-related quality of life measures: literature review and proposed guidelines. J Clin Epidemiol.

